# Childhood adversity and risk of later labor market marginalization in young employees in Sweden

**DOI:** 10.1093/eurpub/ckac131.193

**Published:** 2022-10-25

**Authors:** E Björkenstam, M Helgesson, E Mittendorfer-Rutz

**Affiliations:** 1 Department of Clinical Neuroscience, Karolinska Institutet, Stockholm, Sweden; 2 Department of Community Health Sciences, University of California Los Angeles, Los Angeles, USA; 3 Department of Medical Sciences, Neuroscience, Uppsala University, Uppsala, Sweden

## Abstract

**Background:**

The present study examined the independent and combined effects of childhood adversity (CA) and occupational class on the risk of future labor market marginalization (LMM) in young employees in Sweden. Occupational class (non-manual/manual workers) was also explored as a potential mediator.

**Methods:**

This population-based longitudinal cohort study included 556,793 employees, 19-29 years, residing in Sweden in 2009. CAs included parental death, parental mental and somatic disorders, parental separation, household public assistance, single-parent household and residential instability. Measures of LMM included long-term unemployment (LTU), long-term sickness absence (LTSA) and disability pension (DP). Estimates of risk of each LMM measure, between 2010 and 2016 were calculated as Hazard Ratios (HR) with 95% confidence intervals (CI), using a Cox regression analysis.

**Results:**

Those exposed to CA had an elevated risk for all measures of LMM. Manual workers with a history of household public assistance had the highest risk estimates compared to non-manual workers with no CAs (adjusted HR spanning from 1.59 (LTSA) to 2.50 (LTU). Regardless of occupational class, the risk of LMM grew higher with increasing number of CAs (e.g. adjusted HR of LMM in manual workers with 3+ CAs: 1.87, 95% CI: 1.81-1.94). These patterns persisted after adjustments for a range of confounders, including psychiatric and somatic morbidity. Last, we found a small but significant mediating effect of occupational class in the association between CA and LMM.

**Conclusions:**

Information on CAs are important determinants of LMM in young adults, and especially in manual workers.

**Key messages:**

• Those exposed to childhood adversity had an elevated risk of labor market marginalization, in terms of long-term unemployment, long-term sickness absence and disability pension.

• Information on childhood adversity is an important determinant of labor market marginalization in young adults, and especially in manual workers.

